# The Surgical Outcomes of Modified Intraocular Lens Suturing with Forceps-Assisted Haptics Extraction: A Clinical and Basic Evaluation

**DOI:** 10.3390/jcm13185522

**Published:** 2024-09-18

**Authors:** Yasuyuki Sotani, Hisanori Imai, Maya Kishi, Hiroko Yamada, Wataru Matsumiya, Akiko Miki, Sentaro Kusuhara, Makoto Nakamura

**Affiliations:** 1 Department of Surgery, Division of Ophthalmology, Kobe University Graduate School of Medicine, 7-5-2 Kusunoki-cho, Chuo-ku, Kobe 650-0017, Japan; 2 Department of Ophthalmology, Kansai Medical University, 2-5-1, Shin-machi, Hirakata 573-1191, Japan

**Keywords:** modified intraocular lens suturing, forceps assisted, haptic extraction, surgical outcomes

## Abstract

**Background/Objectives:** Postoperative intraocular lens (IOL) tilt is a risk associated with IOL scleral fixation. However, the cause of IOL tilt during IOL suturing remains unclear. Therefore, this study aimed to evaluate the surgical outcomes of a modified IOL suturing technique and investigate the factors contributing to postoperative IOL tilt and decentration. **Methods:** We included 25 eyes of 22 patients who underwent IOL suturing between April 2018 and February 2020. A modified IOL suturing technique that decreased the need for intraocular suture manipulation was used. Factors contributing to IOL tilt and decentration were investigated using an intraoperative optical coherence tomography (iOCT) system. **Results:** The mean postoperative best-corrected visual acuity improved from 0.15 ± 0.45 to −0.02 ± 0.19 (*p* = 0.02). The mean IOL tilt angle at the last visit after surgery was 1.84 ± 1.28 degrees. The present study reveals that the distance of the scleral puncture site from the corneal limbus had a stronger effect on IOL tilt; meanwhile, the suture position of the haptics had a greater effect on IOL decentration. **Conclusions:** The modified IOL suturing technique, which avoids intraocular suture handling, had favorable surgical outcomes with improved postoperative visual acuity and controlled IOL tilt and decentration. Accurate surgical techniques and careful measurement of distances during surgery are crucial for preventing postoperative IOL tilt and decentration.

## 1. Introduction

The incidence of crystalline and intraocular lens (IOL) dislocations has increased, leading to an increased number of patients requiring IOL scleral fixation [1]. Among the various methods used for IOL scleral fixation, intrascleral IOL fixation and IOL suturing are commonly used [2,3,4]. Intrascleral IOL fixation, specifically the Yamane intrascleral fixation technique, has gained popularity owing to its minimally invasive nature and procedural simplicity [5,6]. Nevertheless, the significance of IOL suturing as a surgical option remains unchanged, specifically in cases with suturing being necessary, especially those with thin sclera and refixation of dislocated IOLs [4]. Therefore, surgeons should be proficient in IOL suturing.

One reason for the decrease in the number of IOL suturing procedures performed is complications related to suturing, including suture loosening, breakage, and exposure outside the sclera [7,8,9]. Additionally, difficulty in the intraocular manipulation of sutures is considered another contributing factor. Consequently, if the need for intraocular suture manipulation during IOL suturing is eliminated and other aspects of the procedure become as straightforward as intrascleral IOL fixation, IOL suturing could become a more accessible choice for many surgeons.

Moreover, postoperative IOL tilt, decentration, and the resulting increase in refractive errors and coma aberrations are unresolved problems in all types of IOL scleral fixation techniques [10,11]. In recent years, the misalignment of a 30-gauge needle during Yamane intrascleral IOL fixation has been reported to be a significant contributing factor to postoperative IOL tilt [12]. However, the cause of IOL tilt during IOL suturing remains unclear. Therefore, investigating the underlying causes of postoperative IOL tilt during suturing is essential.

Therefore, in this study, we investigated the clinical evaluation of the surgical outcomes of modified IOL suturing. Furthermore, as a basic evaluation, we investigated the factors that affect IOL tilt using an intraoperative optical coherence tomography system (iOCT) (RESCAN 700; Carl Zeiss Meditec, Oberkochen, Germany).

## 2. Materials and Methods

### 2.1. Clinical Evaluation

This study included 25 eyes from 22 patients who underwent IOL suturing at our hospital between April 2018 and February 2020. The participants were observed for at least 12 months after surgery. The medical records were retrospectively analyzed. This study was conducted in accordance with the Declaration of Helsinki and approved by the Ethics Committee of Kobe University Graduate School of Medicine (No. B210074, 25 June 2021). The requirement for informed consent was waived by the committee due to the retrospective observational design of this study. However, patients could withdraw consent and opt-out of this study at any time via the hospital homepage. The parameters extracted from the medical records and used for statistical analysis included sex, age, preoperative best-corrected visual acuity (BCVA), BCVA at the last visit after surgery, preoperative intraocular pressure (IOP), IOP 1 day after surgery, IOP 1 week after surgery, IOP at the last visit after surgery, preoperative corneal endothelial cell density (CECD), CECD at the last visit after surgery, IOL tilt angle at the last visit after surgery, and the occurrence of intra- or postoperative complications.

### 2.2. Surgical Procedure

**Figure 1:**
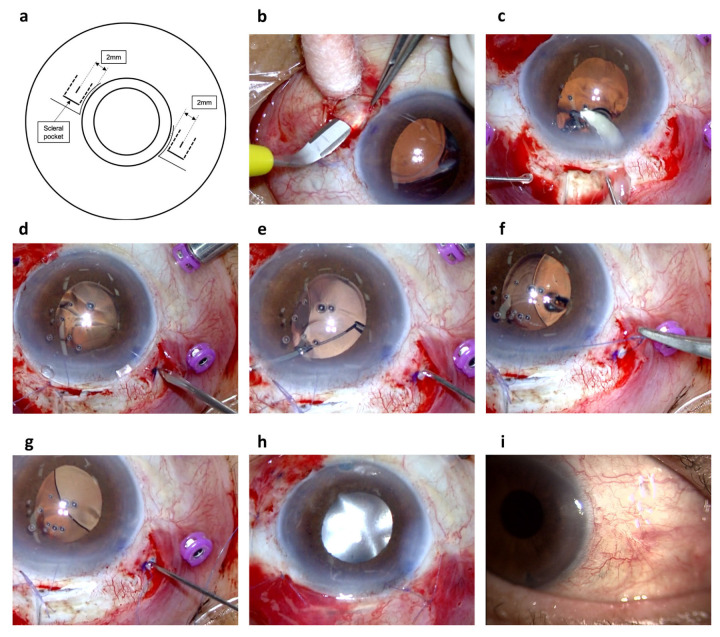
The modified surgical technique for IOL suturing. A schematic diagram of this surgery (**a**). A scleral pocket was created at the same site using a 2.2 mm crescent knife (Mani, Inc., Tochigi, Japan) (**b**). The IOL was resected in half and removed from the main incision (**c**). Scleral incisions were created at the 4 o’clock and 10 o’clock positions on the scleral pockets already created 2 mm away from the corneal limbus using a 20G V-Lance knife (Alcon Surgical, Fort Worth, TX, USA) (**d**). Maxgrip-type ILM forceps (Alcon Laboratories) were inserted into the vitreous cavity from the scleral wound, and the haptics of the IOL were then grasped (**e**) and pulled out for externalization. IOL haptics were sutured using 9-0 polypropylene sutures (Mani, Inc., Tochigi, Japan) and the Cow-hitch technique (**f**). The haptics were grasped with Maxgrip-type ILM forceps and reinserted into the vitreous cavity through the same scleral incision (**g**). The surgery was concluded with suturing of the conjunctiva with 8-0 Vicryl (Ethicon, Raritan, NJ, USA) (**h**). Photographs of the anterior segment 1 year after surgery reveal a smooth conjunctival surface with no exposed conjunctival dissection or suture ligation (**i**).

One of the authors (H.I.) developed this modified IOL suturing technique (see Video S1, Supplemental Digital Content S1, demonstrating the surgical procedure for modified IOL suturing). All surgeries were performed at our hospital by an experienced vitreoretinal surgeon (H.I.). Sub-tenon anesthesia was administered using a 4 mL mixture of 2% lidocaine and 0.5% levobupivacaine. For IOL suturing, the conjunctiva at the 4 o’clock and 10 o’clock positions were incised at the limbus. A scleral pocket was then created at the same site using a 2.2 mm crescent knife (Mani, Inc., Tochigi, Japan) (Figure 1a,b). Next, a 27-gauge pars plana vitrectomy (27GPPV) was performed using a wide-angle non-contact viewing system (Resight^®^; Carl Zeiss Meditec AG, Jena, Germany) with the Constellation Vision System (Alcon Laboratories, Inc., Fort Worth, TX, USA). Three cannulas were inserted through conjunctival displacement and oblique-angled sclerotomies in the inferotemporal, superotemporal, and superonasal quadrants, positioned 3.0–4.0 mm behind the limbus. For eyes without prior vitrectomy, following core vitrectomy, the vitreous gel was visualized by injecting triamcinolone acetonide (MaQaid; Wakamoto Pharmaceutical, Tokyo, Japan) during mid-peripheral vitrectomy. A complete 360° vitrectomy was then performed to remove the peripheral vitreous gel. Posterior vitreous detachment was intentionally induced if necessary. After vitrectomy, for eyes with IOL dislocation, a side-port corneal incision was made using a 20-gauge MVR blade (MVR-Lance; Alcon Laboratories, Inc., Fort Worth, TX, USA). Cohesive viscoelastic material (VISCOAT 0.5; Alcon Japan Ltd., Tokyo, Japan) was injected into the anterior chamber, and the IOL was positioned in the anterior chamber. For eyes with an acrylic IOL, a 2.4 mm bent transconjunctival single-plane sclerocorneal or corneal incision was created using a 2.4 mm slit knife (MSR24; Mani, Inc., Tochigi, Japan) at the 11 o’clock position. The IOL was then bisected and extracted through the primary incision. For eyes with a poly methyl methacrylate IOL, an L-shaped scleral incision (3 mm × 3 mm) or frown scleral incision (7 mm) was created using a 2.2 mm crescent knife (Mani, Inc., Tochigi, Japan) and a 2.4 mm slit knife (MSR24; Mani, Inc., Tochigi, Japan) at the 11 o’clock position. The IOL was then retrieved through the primary incision.

For eyes with a dislocated crystalline lens, if the lens was at the bottom of the fundus, it was made to float to the level of the iris using perfluorocarbon liquid before being removed by phacoemulsification. If the crystalline lens could be stabilized with a capsule expander, it was removed via phacoemulsification and aspiration.

Next, scleral incisions were made at the 4 and 10 o’clock positions on the scleral pockets, 2 mm away from the corneal limbus, using a 20G V-Lance knife (Alcon Surgical, Fort Worth, TX, USA) (Figure 1d). Ophthalmic viscoelastic devices (Healon^®^, Johnson and Johnson Vision, Tokyo, Japan, or Opelead^®^, Senju Pharmaceutical Co. Ltd., Osaka, Japan) were injected to fill the anterior chamber. A foldable acrylic IOL (VA-70 AD; HOYA, Tokyo, Japan) was then inserted into the anterior chamber. Maxgrip-type ILM forceps (Alcon Grieshaber AG; Schaffhausen, Switzerland) were inserted into the vitreous cavity from the scleral wound, and the haptics of the IOL were grasped (Figure 1e) and pulled out for externalization. The IOL haptics were sutured with 9-0 polypropylene sutures (Mani, Inc., Tochigi, Japan) using the Cow-Hitch technique (Figure 1f). The haptics were grasped using MaxGrip ILM forceps (Alcon Grieshaber AG; Schaffhausen, Switzerland) and reinserted into the vitreous cavity through the same scleral incision (Figure 1g). The same procedure was performed at both the 4 and 10 o’clock positions. After reintroducing both haptics into the vitreous cavity, the sutures outside the scleral pocket were pulled to microscopically adjust the position of the IOL. Subsequently, one end of the looped suture was cut inside the scleral pocket, and the non-needle end was pulled out from the pocket. Subsequently, the needle end was passed through the superficial half-layer of the scleral pocket, threading it from outside the eye into the scleral pocket. When the two sutures emerged from the pocket, they were tied beneath the scleral pocket. Viscoelastic substances were removed by irrigation and aspiration (IA) as much as possible. The cannula was removed, and the scleral wound was checked for vitreous incarceration. Where necessary, any vitreous incarceration was resected. The surgery was concluded by suturing the conjunctiva with 8-0 Vicryl (Ethicon, Raritan, NJ, USA) (Figure 1h).

### 2.3. Basic Evaluation

To investigate the factors contributing to IOL tilt and decentration, the experiments detailed below were conducted using the same method as previously described [12]. In summary, both ends of the IOL haptic were secured to a stand 1 mm from the tip, with the optical surfaces positioned horizontally. The stand was then placed under a surgical microscope (RESCAN 700), maintaining a distance of 13 mm between the haptic tips. The inter-haptic distance was measured using the CALLISTO eye function integrated with the surgical microscope (RESCAN 700). Subsequently, images of the IOL line were captured in both vertical and horizontal directions using iOCT, as iOCT generates images with the anterior surface line of the IOL displaying high intensity in the atmosphere. IOL tilt and decentration were analyzed using the IOL position at each time point as the reference. Video S2, Supplemental Digital Content S2 demonstrates IOL imaging and movement under iOCT during the basic evaluation.

Experiment 1: investigation of the effects of inconsistent distance from the limbus to the haptic suturing position on tilt and decentration.

**Figure 2:**
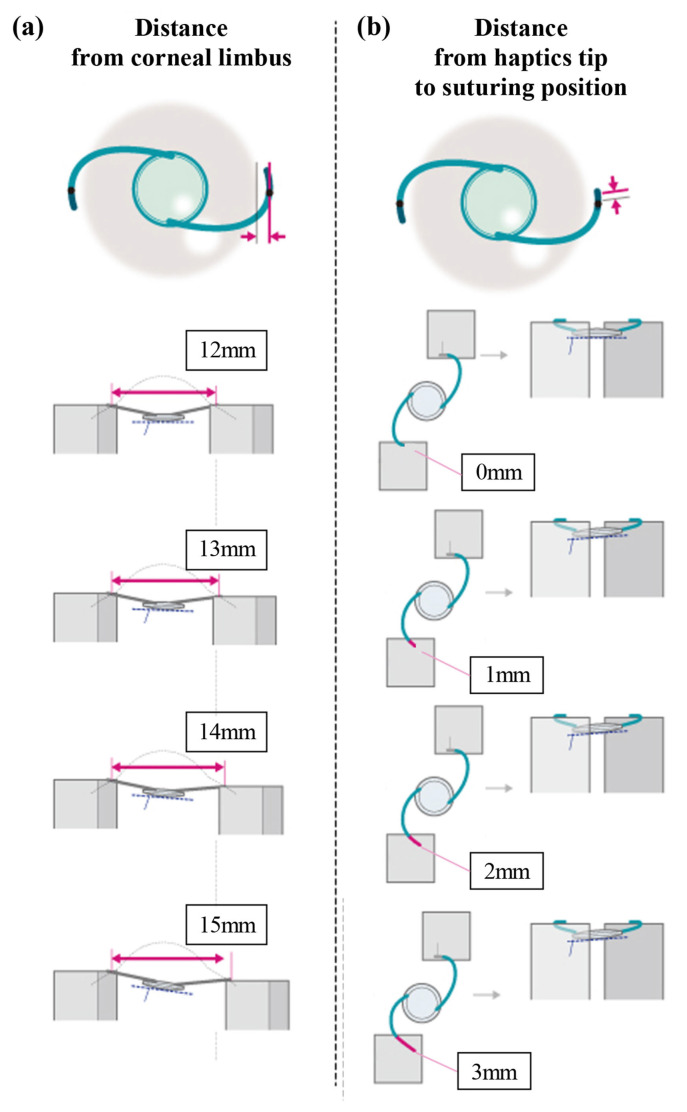
A schematic representation of the experimental evaluations 1 and 2. (**a**) The examination of the effects of varying distances from the limbus to the haptic suturing position on tilt and decentration. One haptic was fixed, and the other haptic was adjusted to maintain distances of 12, 13, 14, and 15 mm between the haptic tips, as observed using CALLISTO photography. (**b**) The examination of the effects of varying distances from the haptic tip to the suturing position on tilt and decentration. Initially, both ends of the IOL haptic were fixed to a stand 1 mm from the tip. Based on this fixed haptic position, one haptic was moved outward by 0, 1, 2, and 3 mm while ensuring that the haptic position remained unchanged.

One haptic was fixed, while the other haptic was adjusted to maintain distances of 12, 13, 14, and 15 mm between the haptic tips, as observed by CALLISTO photography. iOCT and CALLISTO photography were employed to analyze IOL tilt and decentration at each time point (Figure 2a).

Experiment 2: investigation of the effects of inconsistent distance from the haptic tip to the suturing position on tilt and decentration.

As previously mentioned, both ends of the IOL haptic were secured to a stand 1 mm from the tip. The stand was then placed under an operating microscope (RESCAN 700) with a 13 mm distance between the fixed positions of the haptic. Based on the fixed position of the haptic at this point, one haptic was moved outward by 0, 1, 2, and 3 mm while ensuring the haptic position remained unchanged. iOCT and CALLISTO photography were used to analyze IOL tilt and decentration at each time point, respectively (Figure 2b).

Each of the above measurements was performed five times using an IOL (VA-70AD + 20.0D), and the values were used for the analysis.

### 2.4. The Method of IOL Tilt and Decentration Evaluation

For the clinical evaluation, images of the IOL in both the vertical and horizontal directions were captured using swept-source anterior segment OCT (AS-OCT) (SS-1000 CASIA; Tomey Corporation, Nagoya, Japan) during the final visit. For the basic evaluation, IOL images in the vertical and horizontal directions were obtained using iOCT (RESCAN 700).

**Figure 3:**
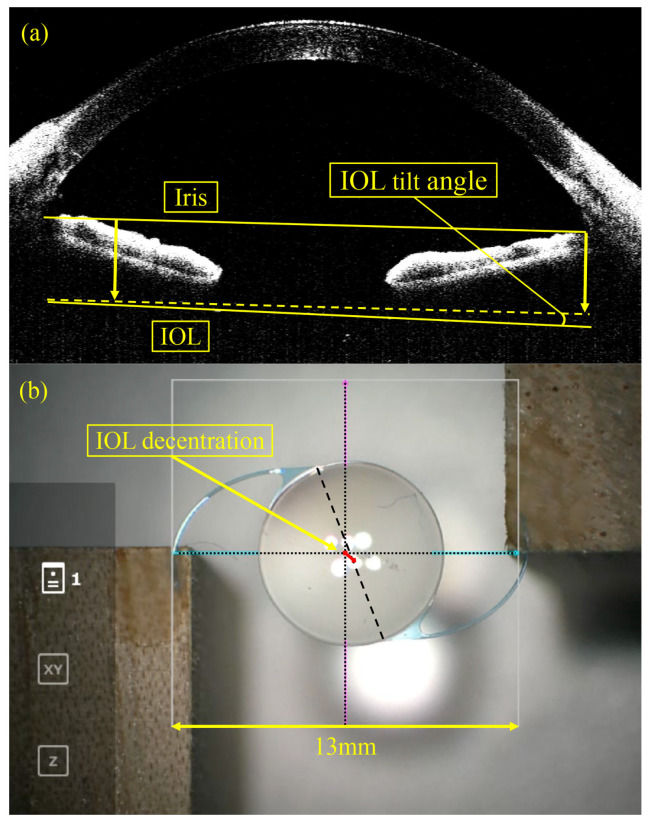
In the clinical evaluation, OCT images were used to measure IOL tilt (**a**). A reference line was drawn through the iris stroma on both sides, and the angle between this reference line and the IOL anterior surface line was defined as the IOL tilt angle. For the experimental evaluation, RESCAN700 images were utilized to measure IOL decentration (**b**). The midpoints of the top, bottom, left, and right lines were connected using a line tool (dotted lines), and the intersection of the two midlines was designated as the center of the frame. The glued parts of the IOL haptics were then connected with a straight line (dashed line), with the midpoint of this line considered the center of the IOL. The length of the measured line segment, or decentration distance (mm), was calculated by the ratio of the line segment length to the frame line length (pt), which corresponds to 13 mm.

To assess IOL tilt, image software (Adobe Photoshop^®^ ver. 25.12.0; Adobe, San Francisco, CA, USA) was employed. IOL images were imported in JPEG format and enlarged using the magnification tool (“Fit on screen”). A reference line was utilized. For clinical evaluation, a line was drawn through the iris stroma on both sides using the “line tool”. A similar line was then drawn along the anterior surface of the IOL using the same tool. The angle between the reference line and the IOL anterior surface line was defined as the IOL tilt angle (Figure 3a). In the basic evaluation, the horizontal direction of the screen served as the reference line, and the inclination of the IOL anterior surface line was measured as the IOL tilt angle. The mean IOL tilt angle in the horizontal and vertical directions was used for analysis.

In the basic evaluation, Adobe Photoshop^®^ ver. 25.12.0 (Adobe, San Francisco, CA, USA) was used to measure IOL decentration. JPEG images of the IOL were imported and enlarged using the magnification tool (“Fit on screen”). The midpoints of the lines (top, bottom, left, and right) were connected with a line tool to determine the center of the frame at the intersection of these midlines. A straight line was drawn connecting the glued parts of the IOL haptics, with the midpoint of this line considered the center of the IOL. The decentration distance (mm) was determined by calculating the ratio of the length of this line segment to the frame line length (pt), which corresponds to 13 mm. The IOL decentration distance (mm) was computed from this decentration distance under basic conditions and for each specific condition (Figure 3b).

For all variables, we report the mean values and standard deviations (SDs). BCVA was converted to the logarithmic minimum angle of resolution (logMAR) for statistical analysis. Friedman’s test and the Wilcoxon *t*-test with Bonferroni correction for post hoc tests were performed to assess changes in IOP, IOL tilt, and decentration. The Mann–Whitney U-test and the Kruskal–Wallis test for continuous variables were used to compare parameters between the groups. Statistical analyses were performed using SPSS software (version 24.0; IBM Corporation, Armonk, NY, USA). Statistical significance was set at *p* < 0.05.

## 3. Results

### 3.1. Clinical Evaluation

Table 1 summarizes the perioperative demographic data of the patients. This study included 13 men and 9 women, with a mean age of 60.0 ± 12.5 years. All patients were followed for at least 12 months post-surgery, with a mean follow-up period of 23.5 ± 5.7 months. The mean preoperative BCVA (logMAR) and BCVA at the final visit after surgery were 0.15 ± 0.45 and −0.02 ± 0.19, respectively (*p* = 0.02). The mean preoperative IOP (mmHg), the IOP on the first day after surgery, the IOP one week post-surgery, and the IOP at the final visit were 17.3 ± 5.8, 15.0 ± 7.3, 16.9 ± 5.8, and 15.7 ± 3.5, respectively (*p* = 0.33). The mean preoperative CECD (cells/mm^2^) and the CECD at the last visit after surgery were 2075 ± 755 and 1695 ± 682, respectively (*p* = 0.07) (Table 1). The mean IOL tilt angle (°) at the last postoperative visit was 1.84 ± 1.28. Postoperative complications included one case of anterior chamber hemorrhage, two cases of vitreous hemorrhage, one case of high intraocular pressure, one case of macular edema, and one case of IOL iris capture. All cases of intraocular hemorrhage were mild and resolved within one week. The other complications improved with conservative treatment.

### 3.2. Basic Evaluation

**Figure 4:**
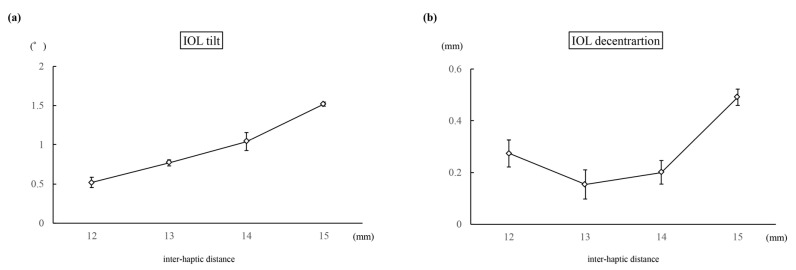
The graph presents the mean IOL tilt (**a**) and the IOL decentration (**b**) when the inter-haptic distance was 12, 13, 14, and 15 mm.

**Figure 5:**
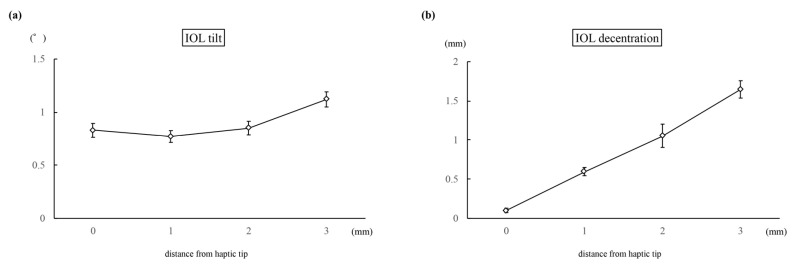
The graph presents the mean IOL tilt (**a**) and the IOL decentration (**b**) when the distance from the haptic tip to the suturing position was 0, 1, 2, and 3 mm.

In Experiment 1, the average IOL tilt angle (°) was 0.52 ± 0.07, 0.77 ± 0.04, 1.04 ± 0.11, and 1.52 ± 0.03 for inter-haptic distances of 12, 13, 14, and 15 mm, respectively (*p* < 0.01) (Figure 4a). The IOL decentration (mm) values were 0.27 ± 0.05, 0.15 ± 0.06, 0.20 ± 0.05, and 0.49 ± 0.03 for the respective inter-haptic distances of 12, 13, 14, and 15 mm (*p* < 0.01) (Figure 4b). In Experiment 2, the average IOL tilt angle (°) was 0.83 ± 0.07, 0.77 ± 0.05, 0.85 ± 0.06, and 1.12 ± 0.07 for distances of 0, 1, 2, and 3 mm from the haptic tip to the suturing position (*p* < 0.01) (Figure 5a). The IOL decentration (mm) values were 0.10 ± 0.03, 0.59 ± 0.05, 1.05 ± 0.15, and 1.65 ± 0.11 for distances of 0, 1, 2, and 3 mm from the haptic tip to the suturing position, respectively (*p* < 0.01) (Figure 5b).

## 4. Discussion

For the clinical evaluation, we assessed the effectiveness of our modified IOL suturing technique and obtained favorable results. In this study, our focus was on two main aspects: the incorporation of a forceps-assisted haptic extraction technique into IOL suturing and the minimization of intraocular manipulation of sutures. Owing to the increasing popularity of intrascleral IOL fixation techniques in recent years [5,6], it is likely that many surgeons are familiar with the use of forceps to manipulate IOL haptics. Implementing the same approach for IOL suturing has several significant advantages. The technique developed in this study obviates the need for intraocular suturing by externally pulling IOL haptics through a scleral wound, similar to the intrascleral IOL fixation technique. We believe that we successfully introduced the method of using forceps to bring the IOL haptic out of the sclera into the IOL suture. Additionally, we addressed the concerns associated with intraocular suture manipulation during the procedure. By extracting IOL haptics from the scleral wound to the external surface of the sclera, we can manipulate sutures outside the sclera, resulting in a safer and more reliable surgical procedure. Notably, this technique does not reduce the incidence of suture-related complications. However, as it facilitates familiar operations (forceps-assisted haptic extraction) and a simpler approach (manipulation of sutures outside the sclera), it can be considered a procedure that contributes to the overall safety improvement of IOL suturing.

Surprisingly, even with this new modified technique, the postoperative mean IOL tilt was 1.84° ± 1.28°. According to previous reports, the IOL tilt after IOL suturing has been reported to be approximately 2.83° to 6.35° [13,14,15]. Several reports have suggested that this tilt is significantly larger compared to that of intrascleral IOL fixation [4,16,17]. However, recent comparative studies have revealed no significant difference in postoperative IOL tilt and decentration between the two techniques [18,19,20]. Our experience, the above results of the clinical evaluation, and the discussions in previous reports support the hypothesis that accurate surgical techniques may effectively control postoperative IOL tilt and decentration, even in cases of IOL suturing; however, the various factors that are particularly important in preventing postoperative IOL tilt and decentration are still unknown. To address these clinical questions, we conducted a basic evaluation of the factors contributing to postoperative IOL tilt and decentration during IOL suturing. The results of the two experiments suggest that the distance of the scleral puncture site from the corneal limbus may have a stronger influence on IOL tilt than the suture position of the haptics. Conversely, the suture position of the haptics is likely to have a greater impact on IOL decentration. The change in the distance from the corneal limbus of the scleral puncture site affects the tilt of the IOL in the anterior–posterior direction of the eyeball. However, the change in the suture position of the haptics essentially includes only a vector in the same plane as the IOL, making it less likely to affect the tilt. These results suggest that in IOL suturing surgery, marking and measuring the distances at the start of the operation are important. If IOL tilt is confirmed during surgery, checking the distance from the corneal limbus of the scleral puncture site and re-puncturing at an appropriate distance may be effective in improving IOL tilt.

This clinical evaluation has some limitations as it is retrospective in nature and subject to selection bias. Additionally, the number of cases was limited. Therefore, the results of this study may not be generalizable to all cases of IOL suturing. Consequently, future prospective studies with large sample sizes are needed. Moreover, in the basic evaluation, the model did not perfectly mimic the clinical conditions. Therefore, the results of our basic evaluation should be considered a reference for assessing the factors contributing to IOL tilt during IOL suturing.

In conclusion, we reported the surgical outcome of a modified technique for IOL suturing that can be performed without intraocular suture handling by externally pulling the IOL haptics through the sclera and assessed the factors that contribute to IOL tilt and decentralization in IOL suturing. We consider this technique to be versatile and worthy of further consideration.

## Figures and Tables

**Table 1:** Perioperative demographic data.

	Before Surgery	Last Visit	*p*-Value
BCVA (logMAR)	0.15 ± 0.45	−0.02 ± 0.19	0.02
IOP (mmHg)	17.3 ± 5.8	15.7 ± 3.5	0.33
CECD (cells/mm^2^)	2075 ± 755	1695 ± 682	0.07

BCVA, best-corrected visual acuity; logMAR, logarithm of the minimum angle of resolution; CECD, corneal endothelial cell density.

## Data Availability

The datasets generated and/or analyzed in the current study are available from H. Imai, the corresponding author, upon reasonable request.

## Supplementary Materials

The following supporting information can be downloaded at https://www.mdpi.com/article/10.3390/jcm13185522/s1, Video S1, Supplementary Digital Content S1: A video demonstration of the surgical procedure of the modified IOL suturing technique; Video S2, Supplementary Digital Content S2: A video demonstration of how the IOL was imaged and moved under iOCT during the basic evaluation.
